# Extracting structured data from unstructured breast imaging reports with transformer-based models

**DOI:** 10.3389/fdgth.2025.1718330

**Published:** 2026-01-09

**Authors:** Mikel Carrilero-Mardones, Jorge Pérez-Martín, Francisco Javier Díez, Iñigo Bermejo Delgado

**Affiliations:** 1 Department of Artificial Intelligence, Universidad Nacional de Educacion a Distancia (UNED), Madrid, Spain; 2 Data Science Institute, Hasselt University, Hasselt, Belgium

**Keywords:** BI-RADS, breast cancer, generative models, BERT models, breast imaging, classification, extractive question answering, structured reporting

## Abstract

**Background and objective:**

Structured clinical data is essential for research and informed decision-making, yet medical reports are frequently stored as unstructured free text. This study compared the performance of BERT-based and generative language models in converting unstructured breast imaging reports into structured, tabular data suitable for clinical and research applications.

**Methods:**

A dataset of 286 anonymised breast imaging reports in Spanish was translated into English and used to evaluate five transformer-based models pre-trained in medical data: BlueBERT, BioBERT, BioMedBERT, BioGPT and ClinicalT5. Two natural language processing approaches were explored: classification of 19 categorical variables (e.g. diagnostic technique, report type, family history, BI-RADS category, tumour shape and margin) and extractive question answering of four entities (patient age, patient history, parenchymal distortion or asymmetries, and tumour size). Multiple fine-tuning strategies and input configurations were tested for each model, and performance was evaluated using accuracy and macro F1 scores.

**Results:**

BioGPT demonstrated the best performance in classification tasks, achieving an overall accuracy of 96.10% and a macro F1 score of 90.30%. This was significantly better than BERT-based models (p=0.012 for accuracy and p=0.017 for F1), particularly in underrepresented categories such as tumour descriptors. In extractive question answering tasks, BioGPT achieved an average accuracy of 93.24%, which is slightly lower than that of BioMedBERT and ClinicalT5, but not significantly so. Notably, BioGPT could perform classification and extractive question answering simultaneously, which is a capability unavailable in BERT-like models.

**Conclusions:**

Generative models, particularly BioGPT, offer a robust and scalable approach to automating the extraction of structured information from unstructured breast imaging reports. Their superior performance, combined with their ability to handle multiple tasks concurrently, highlights their potential to reduce the manual effort required for clinical data curation and to enable the efficient integration of imaging data into research and clinical workflows.

## 1 Introduction

Breast cancer is the most common cancer in women, with an estimated 2.3 million cases worldwide in 2022 [1]. The 5-year survival rate is 90% overall; it rises to 99% if the cancer is detected at an early stage,^1^ but falls to 86% if it has spread to regional lymph nodes and to 29% if it has spread further. Early detection is therefore of paramount importance.

Several techniques are used to diagnose breast cancer, mammography being the most effective screening method. However, its sensitivity decreases in fibroglandular breasts. In these cases, ultrasound can complement mammography by detecting nodules that may have been missed or by providing more detail about a suspicious area identified on the mammogram.

Clear and consistent reporting of imaging findings is essential for accurate diagnosis, effective communication among healthcare providers and optimal patient care [2]. To this end, the American College of Radiology proposed the Breast Imaging Reporting and Data System (BI-RADS) in 1993, ensuring consistent terminology, assessment and follow-up recommendations. The 5th and most recent edition of BI-RADS, published in 2013, introduced support for ultrasound and magnetic resonance imaging (MRI) reporting [3]. Although standardisation facilitates storing data in a structured way that could allow its use for research, most data in hospitals is only stored in free-text medical reports. Furthermore, despite there have been many attempts to standardise and structure these reports, adoption has failed due to concerns about workflow and productivity [4]. For research purposes and more efficient clinical settings, it is important to clean and structure the data, which is a time-consuming task that is often not affordable and may not even be feasible for large datasets.

To help with this task, natural language processing (NLP) has gained popularity in recent years, with the aim of predicting patient outcomes, augmenting hospital triage systems and generating diagnostic models for early disease detection [5]. Reichenpfader et al. [6] conducted a comprehensive review of large language model (LLM) approaches for extracting information from radiology reports. They identified only 34 studies up to August 2023, of which just two focused on breast imaging. Similarly, Saha et al. [7] conducted a scoping review on the application of NLP in breast cancer radiology reports, cataloguing 44 studies published between 1997 and 2022. However, the rapid advancement of LLM architectures means that more capable models are now available. Notably, Lee et al. (2025) analysed 69 studies that employed LLMs for radiology report structuring and reported that prompt engineering significantly improved model accuracy across modalities [8].

Since Bidirectional Encoder Representations from Transformers (BERT) were proposed in 2019 [9], achieving state-of-the-art results in a range of NLP tasks, most natural language problems have been solved using this or similar architectures. However, due to the technical nature of medical language, direct application of these models usually produces unsatisfactory results [10]. For this reason, BERT models have been adapted to medical corpora by pre-training them on domain-specific data, thereby improving their performance. Some of these models are BlueBERT [11], BioBERT [10] and BioMedBERT [12].

Prior work in structuring breast cancer reports using NLP includes Kuling et al. [13], who trained a BERT model to segment the reports into sections such as “title,” “patient history,” “prior imaging reference,” etc. They then extracted modality/procedure, previous cancer, purpose of examination, menopausal status, density and background parenchymal enhancement from these sections using a classifier consisting of a BERT-based encoder followed by a dense neural network classifier. Their model outperformed both classic BERT and BioClinical BERT [14] in both segmentation and classification. In their recent work, Reichenpfader et al. [15] applied BERT-like models that had been further pre-trained with frame semantics in order to extract structured information from German mammography reports. This approach achieved high F1-scores, thereby demonstrating its feasibility. They used a German-language model [medBERT.de [16]], which was initially pre-trained on around 4.7 million medical documents. They then further pre-trained the model using masked language modelling on a corpus of 219,029 radiology reports. The model was then fine-tuned for two tasks: extractive question answering using 210 manually annotated mammography reports and named entity recognition based on a frame semantics schema. To achieve this, the reports were annotated with 14 distinct clinical fact types, each consisting of an “anchor” (the core clinical concept) and associated “modifiers” (additional details such as location, severity or timing). This resulted in a total of 40 entity types (14 anchor types and 26 modifier types).

More recently, generative LLMs have gained popularity, especially since the advent of ChatGPT [17], due to their versatility in different tasks without fine-tuning. As explained for BERT-like models, some of these generative models have been adapted for biomedical data, such as BioGPT [18] and ClinicalT5 [19]. BioGPT is a GPT-2 further pre-trained on 15 million PubMed abstracts and has achieved state-of-the-art results on PubMedQA [20] and better document classification results on the HoC corpus [21] than BioBERT and BioMedBERT. ClinicalT5 is a sequence-to-sequence model further pre-trained on 2 million clinical notes from the MIMIC-III [22] dataset, with initial weights from the SciFive-PubMed-PMC [23] model.

For example, Choi et al. [24] used ChatGPT 3.5 with prompts designed to extract clinical information from surgical pathology and ultrasound breast cancer reports, with time- and cost-efficient results compared to manual annotation. Sanli et al. [25] also used ChatGPT-4o to assign BI-RADS malignancy categories to MRI reports, concluding that ChatGPT can interpret unstructured breast MRI reports. Miaojiao et al. [26] conducted a similar study using breast ultrasound images. In one of their experiments, they provided ChatGPT with BI-RADS descriptions of nodules to classify their malignancy, achieving an accuracy of 80.63%. Liu et al. [27] Used knowledge-driven prompts and Qwen-7b-Chat [28] to extract BI-RADS categories from MRI reports. They demonstrated that with knowledge-based reports they significantly improved the information extraction performance for most categories, but obtained worse results in some of them.

In another instance, Hussain et al. [29] translated Spanish reports into English using Google Translate and compared classical methods (TF-IDF with a classifier), BERT and BioGPT for the task of extracting the BI-RADS malignancy estimate from the reports. BioGPT gave the best results, followed by BERT. However, the comparison might have been unfair, since it included a model further pre-trained on medical data (BioGPT) against one only pre-trained on generic data (BERT).

Our study aims to identify the most effective method of converting free-text breast imaging reports into structured data. Structured data is easier to process for research purposes and can be particularly valuable in clinical settings. For example, it enables the efficient search for patients with specific characteristics and facilitates comparisons between patients with similar findings.

This study makes several novel contributions to the processing of breast imaging reports. Firstly, we compare domain-specific BERT models and medical generative models in classification and extractive question answering tasks. Secondly, we explore various input configurations for each model type. Thirdly, to the best of our knowledge, we are the first to extract ultrasound detailed BI-RADS tumour descriptors, such as shape and margin, directly from free-text reports. We evaluate these models using both cross-validation and testing experiments across 19 classification tasks and four extractive question answering tasks.

## 2 Methods

In this study, we have extracted relevant information from reports using two approaches: classification and extractive question answering. We used classification for entities with a discrete number of possible values or categories, such as the diagnostic technique (e.g., mammography, ultrasound, or both). However, some data could not be categorised; for example, numerical values (e.g., age or size) and free text (e.g., medical history). In these cases, we used extractive question answering, which directly returns the part of the report containing the answer. We begin this section by analysing the various models employed in this study, given that the data preprocessing step is dependent on the type of model.

### 2.1 Models

We compared three main different approaches to modelling.

As a baseline, we initially adopted Bag-of-Words (BoW)-based models, which are limited to classification. These models represent each report using a TF-IDF (Term Frequency-Inverse Document Frequency) vector, which captures the relative frequency of words in the document, weighted by their importance across the entire dataset. We then used a deep neural network with a hidden layer of size 64 and GELU activation function [30] and entity-specific dense layers (i.e., one for diagnostic technique, another for report type, etc.) with softmax activations.

The second approach consisted of BERT-like models. We used the following models further pre-trained on medical data: BioBERT [10], BioMedBERT [12] and BlueBERT [11]. We fine-tuned each model for classification and extractive question answering tasks separately. For classification, we replaced the final layer of the further pre-trained BERT model with a classification head specific to the new task. This head’s input is the pooled [CLS] token, the hidden representation of the special [CLS] token that BERT automatically inserts at the start of every input sequence. During training, BERT learns to encode the overall meaning of the entire input sequence into the [CLS] token. Consequently, its final hidden state captures global contextual information from the entire input. Since BERT was originally trained with this token acting as the input for classification during pre-training (e.g., predicting the next sentence), it has become standard practice to use the [CLS] token representation for subsequent classification tasks. We tried two different architectures for the classification head: one with entity-specific output layers (like in the BoW model) and one with a single output layer of size 63 (the sum of the cardinality of all entities) with a softmax activation function. It is worth noting that the former architecture allows for the classification of all entities at once, whilst the latter architecture allows for one single output at a time. The latter architecture allows for the inclusion of context information in the model input. For example, when extracting “diagnostic technique,” we can append “Additional information: biopsy reports, simple cysts and analysis of lymph or axillary nodes are only seen on ultrasound” to the input. For this approach, it is necessary to specify in the input what entity the model should focus on. More details are given in Section 2.2.2. We fine-tuned the BERT-like models by freezing the pre-trained layers for the first five epochs and unfreezing them for the final four to eight epochs.

The core architecture of the BERT models remains consistent for both extractive question answering and classification tasks, with only the task-specific output layers differing. In the case of question answering, a single question is posed for each clinical report. For example, the question could be “Does the patient’s age appear in the following breast medical report?” The model is fine-tuned to predict the start and end positions of the answer within its context. To achieve this, the task-specific head operates on the hidden states of all the tokens in the input sequence. Two parallel token-level classifiers are applied: one for predicting the start token and one for the end token of the answer. This structure also allows information to be added to the reports.

In the third and final approach, we explored the use of generative models such as BioGPT [18] and ClinicalT5 [19]. These models can be fine-tuned to perform both tasks—extractive question answering and classification—simultaneously. BioGPT is a decoder model trained to generate the next token based on the prior tokens, while ClinicalT5 is an encoder-decoder model. For BioGPT we tried two different fine-tuning methods:

• A two-stage fine-tuning procedure: first, we fine-tuned the models to generate the next word in the report, allowing them to learn the structure and semantics of the language. Second, we fine-tuned the models to generate the next word in the response, focusing specifically on the final classification or extractive question answering task.
• A one-stage fine-tuning procedure: we skipped the first stage and directly fine-tuned the model to produce the answer. This was the fine-tuning method used in ClinicalT5, since this model does not accept the two-stage fine-tuning procedure.

More information on the model fine-tuning parameters can be found in Appendix 1.

### 2.2 Data preparation

In collaboration with two hospitals in Madrid (HM Montepríncipe and HM Vélazquez), we obtained 286 breast imaging reports in Spanish corresponding to 250 patients. Hospital technicians only extracted written medical reports and all personally identifiable information (names, identification numbers, and dates) was removed before extraction, with ethical approval from HM Hospitals. The authors received the information already anonymized and could not be re-identified by them, as they did not have access to the hospital database.

#### 2.2.1 Data cleaning and labelling

Before analysing the data, we divided them into 216 for cross-validation and 70 for testing (approximately 75% of the total samples for fine-tuning and 25% for testing), avoiding introducing reports from the same patient in both datasets. Test data was exclusively used for estimating the performance of the final models.

Reports could refer to ultrasound, mammography (2D, tomosynthesis and both) or both. There were biopsy reports, nodal staging ultrasound reports and more general reports, such as screening or 6-month follow-up over a nodule. The reports followed different structures. Some could be divided into sections on diagnostic tools, reason for consultation, results and conclusions, sometimes clearly distinguished by headings. Other reports had the same structure repeated twice, once for mammography and once for ultrasound. However, some of them would have no apparent structure or order and would mix different parts in the same sentence, making it difficult to divide the task into segmentation and classification, as in Kuling et al. [13].

The reports were automatically pre-processed according to a set of predefined text-cleaning rules, which are described below:

• We created a dictionary of common abbreviations in the field to replace the acronyms with their meaning.
• We removed line breaks, replacing them by a period if the first letter of the word was in uppercase and a space if not (reports often had line breaks in the middle of a sentence).
• We removed punctuation and space errors (double spaces, spaces after punctuations, etc.).
• We converted sentences that were completely uppercase to lowercase.
• We standardised the different ways of writing BI-RADS (BIRADS, birads, bi-rads, etc.) and made some letters uppercase, such as the mammography density (A, B, C, D) or the category in BI-RADS 4 (A, B, C).
• We translated the reports into English to take advantage of the more robust pre-trained models available in this language. For translation of the de-identified reports we used the DeepL API Pro service (DeepL SE, Cologne, Germany). The service is GDPR-compliant, operates under European data-protection regulations and guarantees that submitted texts are not stored or used for model-training.

To label the reports, we first created a rule-based model using regular expressions to extract some of the characteristics: age of the patient, diagnostic technique, type of report, family history, other type of history (previous cancer, biopsy, etc.), having a prosthesis and the final BI-RADS classification. However, these data could only be extracted in the semi-structured reports and it was necessary to supervise, correct and complete some of the results manually. This automated step served to alleviate the manual workload by generating the initial tabular dataset. In a second round, an expert with 4 years of experience in breast cancer research manually annotated the data. We distinguished between mammographic findings and ultrasound findings:

• Mammography: breast density, benign calcifications, suspicious calcifications, lymph nodes, parenchymal distortion or asymmetries, and nodules.
• Ultrasound: breast density, simple cysts, duct ectasia, benign lymph nodes, suspicious lymph nodes and tumours, as well as the BI-RADS descriptors and characteristics mainly used by the radiologists for the tumours: shape, margin, echogenicity, size, if known and stability.

Table 1 shows the 19 categorical variables with their categories and Table 2 shows the four extractive question answering variables with some examples. The imaging modality in which these variables could be found was added to both tables. Mammography density, ultrasound density, shape, margin and echogenicity have the “unknown” label to avoid hallucinations. Since some of the reports do not give these characteristics, fine-tuning only the reports that would result in the model giving one of these outputs for new reports, even if the report does not contain this information. In the extractive question-answering task, we assigned the label “not present” to categories that did not appear in the report, and the models were given this output if they did not find a match.

**Table 1:** Categorical variables used in our study for structured breast imaging reporting.

Variable	Modality	Possible values
Diagnostic technique	–	Mammography; ultrasound; mammography and ultrasound
Report type	–	Biopsy; nodal staging ultrasound; normal control or revision
Family history	–	First degree; second degree; no family history
Prosthesis	–	Yes; no
BI-RADS	–	BI-RADS 0; BI-RADS 1; BI-RADS 2; BI-RADS 3; BI-RADS 4A; BI-RADS 4B; BI-RADS 4C; BI-RADS 5
Mammography density	Mammography	ACR A; ACR B; ACR C; ACR D; unknown
Benign calcifications	Mammography	Yes; no
Lymph nodes	Mammography	Yes; no
Ultrasound density	Ultrasound	Heterogeneous fibroglandular; fibroglandular and fat; homogeneous fibroglandular; homogeneous fatty; unknown
Benign lymph nodes	Ultrasound	Yes; no
Suspicious lymph nodes	Ultrasound	Yes; no
Simple cysts	Ultrasound	Yes; no
Duct ectasia	Ultrasound	Yes; no
Nodules (Ultrasound)	Ultrasound	Yes; no
Shape	Ultrasound (Only if nodule)	Oval; round; irregular; lobulated; unknown
Margin	Ultrasound (Only if nodule)	Circumscribed; not circumscribed; spiculated; indefined; unknown
Echogenicity	Ultrasound (Only if nodule)	Hypoechoic; heterogeneous; complex and cystic; unknown
Known	Ultrasound (Only if nodule)	Yes; no
Stable	Ultrasound (Only if nodule and known)	Yes; grown; shrink

**Table 2:** Examples of extractive question answering.

Variable	Modality	Example of extracted value
Age	–	56
History	–	History of percutaneous excision by means of Vacuum-Assisted Biopsy excision of papilloma of the left breast in 2021
Parenchymal distortion	Mammography	The breast parenchyma shows areas of architectural distortion in the right upper outer quadrant breast adjacent to the coil
Size	Ultrasound (Only if nodule)	13×7×12 mm

#### 2.2.2 Data preprocessing

Data preprocessing was tailored to each model. For the BoW model, the goal was to focus on the most informative words in each report. To achieve this, we first converted all words to lowercase and removed punctuation. Stop words, i.e., articles and prepositions, were also removed, as they do not contribute meaningfully to classification in a BoW representation, which ignores word order and syntax. Next, we applied stemming to reduce words to their root form. We then generated a vocabulary of all the words in the dataset, discarding words that appear only once. Finally, each report was converted to the TF-IDF vector representation. The targets or classification outputs were encoded as one-hot vectors so that the model could be trained with a softmax activation function.

For BERT-like and generative models, we did not modify the reports apart from the initial cleaning. Each model uses its own tokeniser to convert the input report into a sequence of embeddings, i.e., dense vector representations that capture the meaning and context of words. For the model architectures that allow adding information to the input (see Subsection 2.1), we followed the next structure:

• Question: For example, “Question: what diagnostic technique was used in the following breast medical report?”
• Context: For example, “Additional information: biopsy reports, simple cysts and analysis of lymph or axillary nodes are only seen on ultrasound. On the other hand, if the ACR density is given or parenchymal distortions are analysed, the technique will be a mammogram. Tomosynthesis is a type of mammography. The report may include an ultrasound examination, a mammography examination or both.”
• The report.

For the generative models, we added two extra elements to the input.

• Possible answers: explain whether this is a classification or an extractive question answering task and specify the set of valid answers to the question, e.g., “Answer: answer with one of the following options: ‘only ultrasound study,’ ‘only mammography study’ or ‘mammography and ultrasound.”’
• Answer: The correct answer to the question, provided during training. At inference time, only the prefix “Answer:” was included, prompting the model to generate the output.

The size of the input vector in the BERT-like models was set to its maximum of 512, with padding added if the report tokenisation was shorter. However, some of the reports exceeded this limit when context was added. To address this issue, we tried three different techniques:

• No context: keep only the question and the report to avoid truncation.
• Truncate: Add context and simply truncate the report at the maximum length, risking loss of essential information.
• Mixed: include the context in reports where it does not exceed the maximum token limit and leave the remaining reports unchanged.
• Two-stage: fine-tune the initial epochs with the context included and the final epochs without it. This approach allows the model to benefit from the extra data early in fine-tuning, while ensuring that all reports are used in full toward the end.

For the generative models, no truncation was necessary due to less restrictive input criteria. The questions and context used to extract each variable can be found in the code uploaded to GitHub: https://github.com/mikel403/Structuring-Unstructured-Breast-Reports/tree/main.

**Figure 1:**
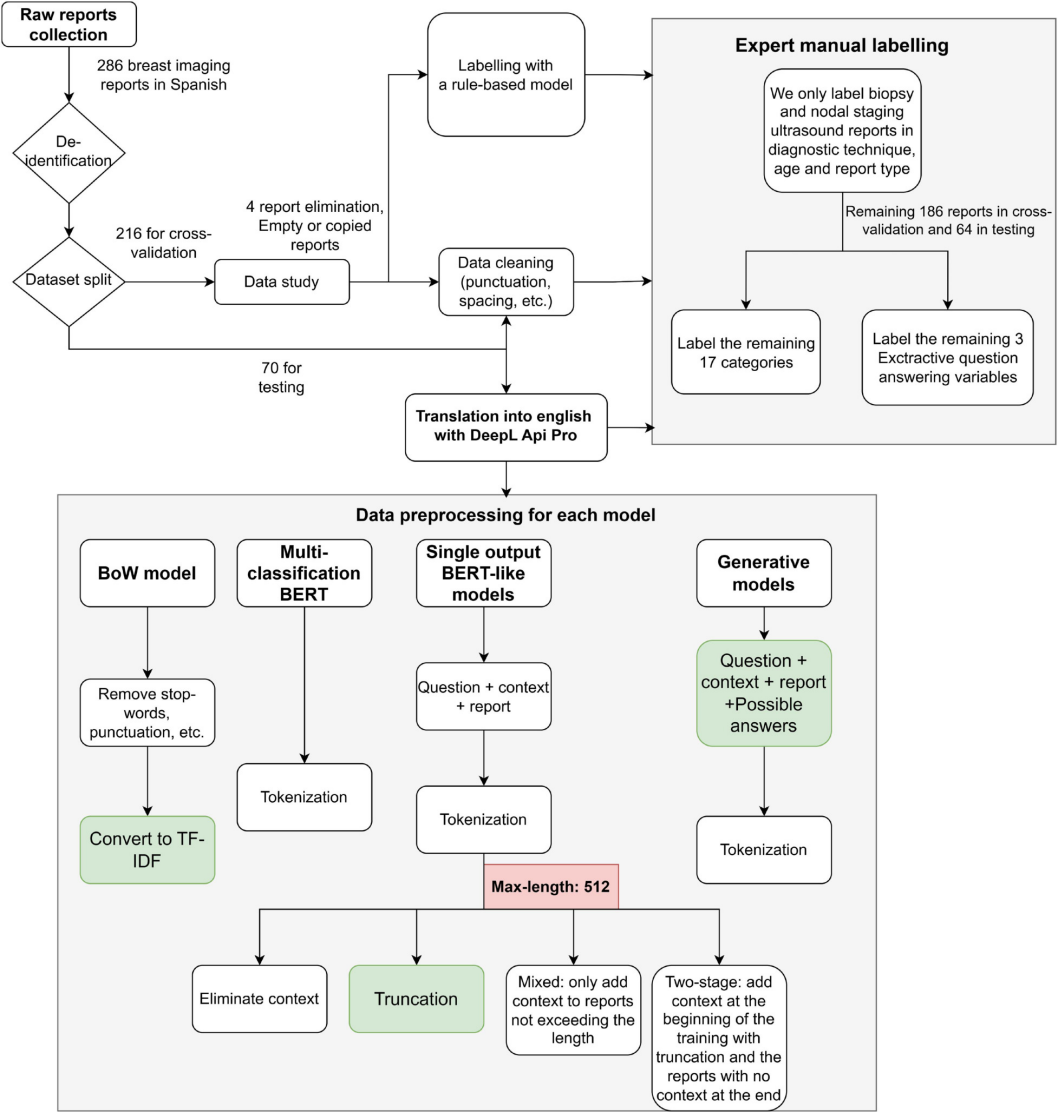
Data preparation pipeline. The main data configuration used in the test experiment after the ablation studies is highlighted in green.

Figure 1 shows a flow chart of the data preparation pipeline, from the data acquisition to the specific configuration for each model.

### 2.3 Experiments

We fine-tuned the models using 216 reports and tested them on the remaining 70. To guide ablation studies, we performed 10-fold cross-validation on the fine-tuning set.

First, we compared two variants of the BioMedBERT model: one with multiple output layers (one per entity) and another with a single output layer, as explained in Section 2.1. For the latter, we studied different ways of dealing with the maximum input length when providing context in the input, as explained in Section 2.2.2.

Next, we compared the two fine-tuning approaches for the BioGPT model explained in Section 2.1 and also studied the effect of the context for the one-stage fine-tuning method.

We then compared the performance of the models selected by the ablation studies with that of the BoW model on the classification tasks. Finally, we compared the generative and BERT-like models on the extractive question answering tasks.

For the classification experiments, we evaluated model performance using both accuracy and the F1 score. As some of the target entities were multi-class rather than binary—such as BI-RADS—we computed the macro F1 score, which ponders all classes equally by taking the unweighted mean of the F1 scores across all classes. This ensures an equal weight for both frequent and infrequent classes. As this is a single-label classification task, accuracy and micro-F1 are equivalent. Therefore, we are reporting micro and macro F1 results.

For many non-binary classification tasks involving descriptors such as shape, margin and echogenicity, we introduced an “unknown” class to account for cases where no descriptor was provided. While this prevents the model from “hallucinating,” it also inflates the accuracy figure due to the large number of “unknown” predictions. In such scenarios, the macro F1 score is particularly valuable as it provides insight into the model’s performance on the minority classes, i.e., the meaningful descriptor categories, rather than being dominated by the majority “unknown” class.

For the extractive question answering task, the outputs were evaluated by the same expert that did the labelling. They manually revised the errors made by the models to determine whether they were genuine errors or minor modifications, such as starting or ending a phrase one word earlier or later, or differences in stop words. If the model provided only part of the output and omitted important information, a score of 0.5 was given. For example, if the expected output was “post-treatment changes in left breast. Asymmetric density in outer quadrants of the right breast, stable” and the output given by the model was “post-treatment changes in left breast.” This was the best method to assess whether the generative models hallucinated. To enhance transparency and reproducibility, we also report the established metric BERTScore [31], which compares the contextual embeddings of the reference and predicted outputs. Unlike exact match metrics, BERTScore accommodates lexical variation while still evaluating whether essential information has been conveyed correctly.

Finally, since it cannot be assumed that the distribution of score differences follows a Gaussian distribution, statistical significance was assessed using the paired Wilcoxon signed-rank test. The unit of analysis was determined by the level at which each metric is defined. In the classification task, although accuracy is available at the instance level, it only takes three discrete values (0, 0.5 and 1), making it unsuitable for the Wilcoxon test. Moreover, F1 is only defined at an aggregated level. For these reasons, comparisons between models were performed at the category level, using one paired score per category across the 19 variables. In contrast, BERTScore provides a continuous value for each individual report–question pair in the extractive QA task. Consequently, comparisons were carried out at the example level, which offers a more appropriate and statistically robust basis for inferential testing. Four variables were defined in this task, but we did not apply a Wilcoxon test at the variable level because the sample size (n=4) was too small.

## 3 Results

### 3.1 Ablation studies and cross-validation results

Table 3 presents the results of the comparison between different configurations of BioMedBERT (described in Section 2.1) in the cross-validation experiment. The models with a single output layer achieved better accuracy than the model with multiple, entity-specific output layers. There were no statistically significant differences in the performance across different approaches to appending the context to our model. However, since truncation gave slightly better results, we used that approach in the test experiment with BERT-like models. We repeated the experiment with BioBERT, reaching the same conclusions.

**Table 3:** Ablation study in the cross-validation experiment: classification accuracy for BioMedBERT with different architectures and approaches to handling maximum input size when providing context.

Entity (number of cases)	Multiple output layers	No context	Two-stage	Hybrid	Truncate
Diagnostic technique (212)	93.40	97.64	**98.11**	97.64	97.17
Report type (212)	97.17	97.17	96.23	97.17	**97.65**
Family history (186)	92.47	**98.92**	**98.92**	**98.92**	97.85
Prosthesis (186)	98.92	**99.46**	**99.46**	**99.46**	**99.46**
BI-RADS (186)	77.42	**96.77**	94.09	95.70	95.70
MMG density (186)	80.11	96.77	97.31	97.31	**98.39**
Benign calcifications (186)	82.80	**98.39**	97.85	96.77	**98.39**
Lymph nodes on MMG (186)	92.47	**98.39**	**98.92**	97.85	97.31
US density (186)	72.58	94.09	91.40	93.01	**94.62**
Benign lymph nodes on US (186)	91.94	95.70	94.62	94.62	**96.26**
Suspicious lymph nodes on US (186)	95.70	97.85	**98.39**	96.77	97.85
Simple cysts (186)	84.95	**97.85**	**97.85**	**97.85**	97.31
Duct ectasia (186)	90.86	**100.00**	**100.00**	**100.00**	**100.00**
Nodules on US (186)	84.95	93.01	**94.62**	**94.62**	93.01
Shape (82)	59.76	89.02	89.02	89.02	**91.46**
Margin (82)	73.17	84.15	80.49	84.15	**86.59**
Echogenicity (82)	69.51	**93.90**	91.46	**93.90**	92.68
Known (82)	70.73	75.61	81.71	**84.15**	82.93
Stable (43)	81.40	81.40	83.72	83.72	**86.05**
**Mean**	83.70	94.00	93.90	94.35	**94.77**

US and MMG are abbreviations for ultrasound and mammography.

Bold values indicate the highest score across the compared models.

Table 4 shows the results for classification with the two different fine-tuning methods for BioGPT and the effect of providing the context (explained in Section 2.2.2) for the cross-validation experiment. Since the one-stage method with context obtained slightly better results, we used this setup for the test experiment.

**Table 4:** Ablation study in the cross-validation experiment: classification accuracy of BioGPT using two training strategies, with and without context.

Entity (number of cases)	No context	One-stage	Two-stage
Diagnostic technique (212)	97.17	**98.11**	**98.11**
Report type (212)	**97.64**	96.70	96.23
Family history (186)	98.39	98.39	**98.92**
Prosthesis (186)	**99.46**	**99.46**	**99.46**
BI-RADS (186)	**98.39**	97.85	**98.39**
MMG density (186)	96.77	97.31	**97.85**
Benign calcifications (186)	98.39	97.31	**99.46**
Lymph nodes in MMG (186)	96.24	**96.77**	**96.77**
US density (186)	**96.74**	95.65	95.11
Benign lymph nodes in US (186)	95.16	**96.24**	94.08
Suspicious lymph nodes in US (186)	**98.92**	97.31	96.24
Simple cysts (186)	97.85	97.85	**98.92**
Duct ectasia (186)	**99.46**	98.92	98.92
Nodules in US (186)	**98.39**	94.62	93.55
Shape (82)	90.24	87.80	**91.46**
Margin (82)	87.80	**91.46**	85.37
Echogenicity (82)	92.68	91.46	**93.90**
Known (82)	75.61	**82.93**	79.27
Stable (43)	88.37	**90.70**	**90.70**
**Mean**	94.93	**95.10**	94.88

US and MMG are abbreviations for ultrasound and mammography.

Bold values indicate the highest score across the compared models.

Tables 5, 6 show a comparison of the selected Transformer-based models with the BoW model in the cross-validation classification experiment. Tables 7, 8 present the results of the extractive question answering experiment. BioGPT achieved the highest mean performance across both tasks and multiple evaluation metrics. Since BioMedBERT was the second best option, we tested the null hypothesis that BioMedBERT performed equally well or better than BioGPT. No statistically significant differences were found, with *p*-values of 0.95 (accuracy) and 0.81 (macro F1) for the classification task and 0.98 for the BERTScore F1 in the extractive question answering task.

**Table 5:** Classification accuracy in the cross-validation experiment.

Entity (number of cases)	BoW	BioBERT	BlueBERT	BioMedBERT	ClinicalT5	BioGPT
Diagnostic technique (212)	94.81	**98.58**	96.70	97.64	97.17	98.11
Report type (212)	96.23	95.28	95.28	**97.17**	95.28	96.70
Family history (186)	95.16	98.39	94.09	**98.92**	93.01	98.39
Prosthesis (186)	98.92	**99.46**	98.92	**99.46**	**99.46**	**99.46**
BI-RADS (186)	81.18	95.16	73.12	95.70	75.81	**97.85**
MMG density (186)	84.41	**97.31**	90.86	**97.31**	96.24	**97.31**
Benign calcifications (186)	77.42	**98.39**	90.32	96.77	97.31	97.31
Lymph nodes in MMG (186)	96.24	**98.39**	96.24	97.85	93.55	96.77
US density (186)	84.95	94.09	81.18	93.01	79.35	**95.65**
Benign lymph nodes in US (186)	91.94	94.62	93.01	94.62	91.40	**96.24**
Suspicious lymph nodes in US (186)	96.24	97.31	95.70	96.77	95.16	**97.31**
Simple cysts (186)	87.63	**98.39**	93.55	97.85	97.31	97.85
Ductal ectasia (186)	96.24	99.46	**100.00**	**100.00**	98.39	98.92
Nodules in US (186)	89.78	91.94	88.71	**94.62**	90.32	**94.62**
Shape (82)	65.85	89.02	74.39	89.02	**90.24**	87.80
Margin (82)	79.27	84.15	84.15	84.15	84.15	**91.46**
Echogenicity (82)	73.17	89.02	89.02	**93.90**	91.46	91.46
Known (82)	78.05	**85.37**	70.73	84.15	78.05	82.93
Stable (43)	86.05	86.05	79.55	83.72	79.07	**90.70**
**Mean**	87.03	94.23	86.09	94.77	90.67	**95.10**

US and MMG are abbreviations for ultrasound and mammography.

Bold values indicate the highest score across the compared models.

**Table 6:** Macro F1 score for classification in the cross-validation experiment.

Entity (number of cases)	BoW	BioBERT	BlueBERT	BioMedBERT	ClinicalT5	BioGPT
Diagnostic technique (212)	62.97	**66.06**	47.00	64.88	64.88	65.67
Report type (212)	78.21	70.56	69.70	**90.19**	62.09	82.06
Family history (186)	79.90	91.41	47.67	**92.54**	84.21	90.77
Prosthesis (186)	94.16	**97.23**	94.16	**97.23**	9**97.23**	**97.23**
BI-RADS (186)	47.49	66.63	23.54	74.86	82.70	**93.41**
MMG density (186)	82.56	93.62	44.23	**98.73**	96.33	97.30
Benign calcifications (186)	77.29	**98.38**	89.75	**98.38**	97.31	97.30
Lymph nodes in MMG (186)	60.15	**88.04**	49.04	80.07	48.33	74.17
US density (186)	81.44	82.47	43.06	91.32	77.80	**92.27**
Benign lymph nodes in US (186)	86.02	90.79	88.92	93.31	87.02	**93.63**
Suspicious lymph nodes in US (186)	86.02	90.02	40.34	**92.28**	85.81	89.28
Simple cysts (186)	87.47	**98.38**	92.95	97.29	97.30	97.83
Duct ectasia (186)	84.99	98.24	96.37	**100.00**	94.72	96.58
Nodules in US (186)	89.65	91.83	86.18	92.92	90.23	**94.58**
Shape (82)	34.94	53.24	29.71	55.40	**66.13**	61.20
Margin (82)	42.75	43.02	28.35	44.65	48.51	**73.33**
Echogenicity (82)	29.49	36.92	25.97	51.15	50.78	**57.08**
Known (82)	77.93	**85.29**	70.57	82.89	77.72	82.92
Stable (43)	40.29	40.29	**59.02**	40.29	29.44	53.80
**Mean**	69.67	78.02	59.02	80.97	75.71	**83.70**

US and MMG are abbreviations for ultrasound and mammography.

Bold values indicate the highest score across the compared models.

**Table 7:** Expert-assessed extractive question answering score in the cross-validation experiment.

Entity (number of cases)	BioBERT	BlueBERT	BioMedBERT	ClinicalT5	BioGPT
Age (212)	**99.53**	**99.53**	**99.53**	**99.53**	**99.53**
History (186)	95.16	94.62	95.43	89.78	**95.70**
Parenchymal distortion (186)	95.16	95.16	95.16	**96.77**	**96.77**
Size (82)	87.80	86.59	91.46	89.02	**92.68**
**Mean**	94.41	93.98	95.67	93.78	**96.17**

Bold values indicate the highest score across the compared models.

**Table 8:** BERTScore F1 and recall for extractive question answering in the cross-validation experiment.

Entity (number of cases)	BioBERT	BlueBERT	BioMedBERT	ClinicalT5	BioGPT
BERTScore F1					
Age (212)	**99.76**	**99.76**	**99.76**	99.10	**99.76**
History (186)	95.76	95.64	**96.28**	91.48	95.98
Parenchymal distortion (186)	95.98	96.37	96.23	96.59	**96.68**
Size (82)	91.05	92.32	94.77	93.71	**95.26**
**Mean**	95.61	96.02	96.76	95.21	**96.92**
BERTScore Recall					
Age (212)	**99.82**	**99.82**	**99.82**	99.39	**99.82**
History (186)	95.15	95.25	95.58	91.89	**95.68**
Parenchymal distortion (186)	95.00	95.47	95.21	**96.56**	95.93
Size (82)	90.84	92.56	**94.66**	93.26	94.63
**Mean**	95.20	95.78	96.32	95.28	**96.52**

Bold values indicate the highest score across the compared models.

### 3.2 Main results

Table 9 shows the comparison between the accuracy of the BoW, BERT-like and generative models on the test data. BioBERT, BioMedBERT and BioGPT outperformed the other models, the latter having a slightly better overall accuracy. Table 10, shows the macro F1 scores, which exhibit the same patterns but with wider performance differences across entities, especially in the BI-RADS tumour descriptors. As in the cross-validation experiment, we tested the null hypothesis that BioMedBERT performed equally well or better than BioGPT. However, with *p*-values of 0.012 for accuracy and 0.017 for macro F1, we can reject this hypothesis and conclude that BioGPT’s performance is statistically superior across the 19 variables in the test experiment. Supplementary Material to this article show the confusion matrices for the BioGPT model’s outputs for each entity in the classification task.

**Table 9:** Main results: accuracy for classification in the test experiment.

Entity (number of cases)	BoW	BioBERT	BlueBERT	BioMedBERT	ClinicalT5	BioGPT
Diagnostic technique (70)	92.86	98.10	95.71	**100.00**	97.14	**100.00**
Report type (70)	**100.00**	97.14	97.14	98.57	94.29	98.57
Family history (64)	93.75	**98.41**	96.88	96.88	92.19	96.88
Prosthesis (64)	**100.00**	**100.00**	**100.00**	**100.00**	**100.00**	**100.00**
BI-RADS (64)	79.69	96.88	71.88	96.88	92.19	**98.44**
MMG density (64)	92.19	96.88	90.63	**100.00**	93.75	**100.00**
Benign calcifications (64)	82.81	95.31	90.63	96.88	95.31	**98.44**
Lymph nodes in MMG (64)	95.31	95.31	95.31	96.88	**98.44**	**98.44**
US density (64)	79.69	93.75	89.06	**98.44**	90.63	**98.44**
Benign lymph nodes in US (64)	89.06	92.19	89.06	87.50	87.50	**93.75**
Suspicious lymph nodes in US (64)	96.88	**98.44**	**98.44**	**98.44**	**98.44**	**98.44**
Simple cysts (64)	79.69	98.44	95.31	98.44	93.75	**100.00**
Duct ectasia (64)	96.88	**98.44**	**98.44**	**98.44**	**98.44**	**98.44**
Nodules in US (64)	85.94	**95.31**	85.94	93.75	87.50	93.75
Shape (25)	56.00	84.00	72.00	72.00	**88.00**	**88.00**
Margin (25)	92.00	88.00	76.00	88.00	80.00	**96.00**
Echogenicity (25)	68.00	88.00	84.00	92.00	**96.00**	**96.00**
Known (25)	76.00	80.00	**84.00**	**84.00**	76.00	80.00
Stable (13)	**92.31**	84.62	69.23	84.62	76.92	**92.31**
**Mean**	86.79	93.67	88.40	93.77	91.39	**96.10**

US and MMG are abbreviations for ultrasound and mammography.

Bold values indicate the highest score across the compared models.

**Table 10:** Main results: macro F1 score for classification in the test experiment.

Entity (number of cases)	BoW	BioBERT	BlueBERT	BioMedBERT	ClinicalT5	BioGPT
Diagnostic technique (70)	90.09	98.10	94.29	**100.00**	97.54	**100.00**
Report type (70)	**100.00**	93.92	92.81	96.71	51.62	96.71
Family history (64)	67.93	74.75	71.16	72.58	**87.67**	69.95
Prosthesis (64)	**100.00**	**100.00**	**100.00**	**100.00**	**100.00**	**100.00**
BI-RADS (64)	51.26	83.72	42.58	83.72	94.03	**95.02**
MMG density (64)	91.57	96.34	90.62	**100.00**	94.15	**100.00**
Benign calcifications (64)	82.81	95.28	90.48	96.86	95.30	**98.43**
Lymph nodes in MMG (64)	48.80	48.80	48.80	74.19	**89.59**	**89.59**
US density (64)	78.33	89.48	86.00	**98.77**	90.40	**98.77**
Benign lymph nodes in US (64)	76.26	78.60	73.57	67.92	74.14	**83.96**
Suspicious lymph nodes in US (64)	74.19	**82.93**	**82.93**	**82.93**	**82.93**	**82.93**
Simple cysts (64)	79.44	98.43	95.28	98.43	93.74	**100.00**
Duct ectasia (64)	74.19	**92.44**	**92.44**	**92.44**	**92.44**	**92.44**
Nodules in US (64)	85.65	**95.17**	85.65	93.52	86.87	93.44
Shape (25)	36.78	64.02	53.33	56.86	**92.00**	89.93
Margin (25)	64.58	62.31	40.37	63.31	42.87	**90.58**
Echogenicity (25)	36.36	69.35	44.51	71.50	**73.81**	**73.81**
Known (25)	75.96	80.00	**83.97**	**83.97**	75.96	80.00
Stable (13)	**81.16**	45.83	40.91	45.83	43.48	**81.16**
**Mean**	73.44	81.55	74.20	83.13	82.02	**90.35**

US and MMG are abbreviations for ultrasound and mammography.

Bold values indicate the highest score across the compared models.

Regarding extractive question answering, Table 11 shows that BioMedBERT achieved the best overall accuracy, assessed by the expert, by a narrow margin over ClinicalT5 and BioGPT, even if each of the models was best in a different entity.

**Table 11:** Main results: expert-assessed extractive question answering score in the test set.

Entity (number of cases)	BioBERT	BlueBERT	BioMedBERT	ClinicalT5	BioGPT
Age (64)	**100.00**	97.14	**100.00**	**100.00**	**100.00**
History (64)	93.75	93.75	**95.31**	87.50	94.53
Parenchymal distortion (64)	95.31	87.50	96.09	94.53	**98.44**
Size (25)	72.00	32.00	84.00	**92.00**	80.00
**Mean**	90.27	77.60	**93.85**	93.51	93.24

Bold values indicate the highest score across the compared models.

Table 12 shows the BERTScore results, which are similar: BioMedBERT obtained best BERTScore F1 by a narrow margin, having better precision, but worse recall than BioGPT, but the difference was not significant (p=0.34).

**Table 12:** Main results: BERTScore F1 and recall for extractive question answering in the test experiment.

Entity (number of cases)	BioBERT	BlueBERT	BioMedBERT	ClinicalT5	BioGPT
BERTScore F1					
Age (64)	**100.00**	98.53	**100.00**	**100.00**	**100.00**
History (64)	94.26	93.86	**95.20**	89.62	94.74
Parenchymal distortion (64)	94.72	89.00	**95.65**	94.24	94.15
Size (25)	88.08	63.73	95.17	**96.03**	94.82
**Mean**	94.27	86.28	**96.51**	95.63	95.91
BERTScore Recall					
Age (64)	**100.00**	98.16	**100.00**	**100.00**	**100.00**
History (64)	92.94	91.58	93.95	89.93	**94.93**
Parenchymal distortion (64)	94.12	87.37	**94.66**	93.69	94.41
Size (25)	89.77	62.07	93.51	**95.65**	94.78
**Mean**	94.21	84.80	95.53	95.19	**95.98**

Bold values indicate the highest score across the compared models.

## 4 Discussion

We have compared the latest BERT and generative models further pre-trained on medical data to extract relevant information from breast imaging (mammography and ultrasound) reports. In addition, we have assessed the performance of these models using different architectures, fine-tuning strategies and approaches to provide context.

We have demonstrated that these models can achieve high accuracy on such tasks, highlighting their potential as tools for extracting valuable information from electronic health records, both for research purposes and for use in efficient clinical workflows.

We experimented with different structures within the BERT-like and generative models in the cross-validation experiment. For BioGPT, there was no significant difference across architectures. However, for the BERT-like models, the architecture that adds questions and context to reports and extracts one answer per report outperformed the structure with multiple output layers achieving a mean accuracy of 94.77% vs. 83.70%. Although adding multiple output layers to the network’s head is the most common approach for multiple classifications, this can make the task difficult for BERT-like models when faced with a large number of classification tasks, as was the case in this study. Since BERT-like models are pre-trained as language models, they benefit from additional context provided in natural language. When a question is added (e.g., “Is the patient’s age mentioned in the report?”), the model focuses on a specific aspect of the input, enabling it to better associate the question with the appropriate output neuron. By contrast, a model with multiple heads processes all tasks simultaneously without explicit task context, which can hinder learning, particularly when tasks are not fully independent. Interestingly, a BoW model using TF-IDF features as input and multiple output layers, achieved a mean accuracy of 87.03%, outperforming the BERT-based multi-output head model. To investigate this further, we conducted an additional experiment in which we put the classification network of the BoW model as the head of the frozen BioMedBERT model. We then trained it on top of the frozen BioMedBERT. This setup achieved an accuracy of 80.26%, suggesting that the classification network favoured the explicit TF-IDF features over the frozen BioMedBERT representations. Only when adding the questions and a single output layer was BioMedBERT able to outperform the BoW model.

The generative BioGPT model outperformed all other models in the classification task (p=0.012), achieving slightly lower scores in the extractive question-answering task, though not significantly so. Furthermore, BioGPT was fine-tuned to perform both tasks simultaneously, whereas BERT-like models could not. The F1 results show a greater difference between this model and the others, meaning that BioGPT also performed better when classifying minority groups. Finally, when examining the BI-RADS descriptors, BioGPT demonstrated a superior grasp of the categories and the various terms associated with them. We also fine-tuned this model to extract tumour localisation, which would not be possible with BERT-like models, as it is often divided into different parts of the sentence. In the test experiment, BioGPT achieved an accuracy of 94.00%; the errors occurred when the tumour location was not fully specified. We also fine-tuned this model for symptomatic extraction. The model obtained an accuracy of 99.22% when considering non-symptomatic cases as a separate class (meaning there was no hallucination problem) and 3.5 out of 4 when considering only symptomatic cases. The model made a partial error: it predicted a palpable nodule, but did not indicate that it was painful.

We added an “unknown” label to mammography and ultrasound density, as well as to shape, margin and echogenicity. Using this label alongside the BioGPT model, we achieved macro F1 scores of 100, 98.77, 89.93, 90.58 and 73.81. When only reports with a BI-RADS label for these variables are considered, i.e., excluding the “unknown” label, the macro F1 scores are 100, 99.33, 93.01, 88.89 and 66.67. For clarity and transparency, the confusion matrix for each variable is available in the Supplementary Material to help the reader better understand the outputs of the model.

We compared the inference times of BioGPT and BioMedBERT using a representative report from the test set and a single NVIDIA V100 GPU with 16 GB of HBM2 memory. BioGPT took 9.57 s to carry out both classification and extractive question answering in a single forward pass. By contrast, BioMedBERT took approximately 1.57 s per task, totalling 3.14 s for both. Although the latter is faster in terms of raw inference time, the fact that BioGPT can perform both tasks simultaneously offers advantages in terms of deployment simplicity, reduced system complexity and potentially improved consistency of outputs. In real-world clinical settings, the importance of these trade-offs depends on the context. In latency-sensitive applications, such as interactive hospital systems where quick responses are essential, smaller, faster models like BioMedBERT may be more suitable, particularly if their performance is comparable. However, in batch processing or retrospective analysis scenarios, where large volumes of data can be processed without real-time constraints, multitask models like BioGPT may be more efficient and convenient.

Previous studies have explored the use of BERT-like and generative models for extracting information from breast medical reports [13, 15, 24, 27, 29], demonstrating the effectiveness of these approaches. However, there are few comprehensive comparisons between generative and BERT-like models. The comparison made by Hussain et al. [29] involved only a single medically further pre-trained model (BioGPT) compared against a general-purpose one (BERT), which limits the scope of the evaluation. Finally, Reichenpfader et al. [15] further pre-trained a BERT model for NER and extractive question answering, with promising results. They compare their results with the open-source Llama 3.3 model, but do not further fine-tune a generative model specialized in medical tasks.

The main limitation of this study is that there were only 286 reports and some of the classes were underrepresented. For instance, while the “mammography and ultrasound” and “ultrasound” techniques were balanced, there were only two “mammography” exams, both of which were included in the cross-validation experiment (in different folds). As can be seen from the macro F1 scores in Table 6, none of the models could correctly label these reports. There was also only one case of third-degree family history in the dataset and this was included in the test set. Only ClinicalT5 could correctly identify it. This is why the macro F1 score, in conjunction with accuracy, provides a clearer picture of the results. Furthermore, the confusion matrices of the test results have been added to the Supplementary Material. These also illustrate the overall balance of the data in our dataset. A larger dataset would have allowed us to demonstrate the differences and similarities between the models more effectively. Nevertheless, this dataset was sufficient for fine-tuning and achieving good results with BioBERT [10], BioMedBERT [12] and BioGPT [18], particularly the latter. Considering that we obtained multiple classifications or extractions from each report, the dataset comprised 5,399 items.

Another limitation is that we only obtained reports from two hospitals located in the same city, belonging to the same hospital group, HM. Therefore, the fine-tuned model could be useful for converting medical reports to tabular data in these hospitals; however, generalisability to other centres was not explored in this study. Additionally, a single expert annotated the reports and assessed the accuracy of the extractive question answering task. While this approach ensured consistency, it might have introduced annotation bias. Future work should therefore incorporate inter-rater agreement to strengthen the reliability of the evaluation. Furthermore, we explicitly distinguished between findings derived from mammography and those from ultrasound. Most reports included both modalities, sometimes clearly separating them and at other times mixing them within the same paragraph. While the differing descriptions across imaging modalities enabled clear labelling during annotation, there is a risk that models might learn incorrect associations and misattribute findings to the wrong modality. In future work, we plan to investigate this potential bias.

Lastly, we note that another limitation is that we did not compare our results with those of general-purpose state-of-the-art (SOTA) LLMs, such as GPT 4 or GPT 5. Although these models have demonstrated excellent performance in a variety of natural language processing tasks, we focused on evaluating domain-specific, open-access models that can be fine-tuned and deployed on local infrastructure to ensure compliance with data privacy requirements.

We compared BERT-like and generative models that had been further pre-trained on medical data in order to automatically convert breast medical reports into tabular data. Tabular data is easier to process for research purposes and can support clinical use by enabling efficient information retrieval and patient comparison. After determining the optimal architecture, fine-tuning strategy and input configuration for each model through cross-validation, we tested them on an additional 70 reports, achieving the best results with the generative BioGPT model. Fine-tuning BioGPT on our medical reports yielded accuracies of 96.10% for classification and 93.24% for extractive question answering, establishing it as a promising tool for reducing the burden of labelling breast medical reports.

Future work will involve fine-tuning the generative model using more anonymised medical reports from a wider variety of hospitals. Due to the power of generative models, we could also analyse the artificial generation of breast medical reports using an online service such as ChatGPT version 5, as done by Reichenpfader et al. [15]. Importantly, these models would be used only for generating artificial data, not for processing real clinical reports, thereby ensuring that no sensitive information is shared. Previous studies have used earlier versions of ChatGPT to label medical data [24–26], demonstrating an adequate grasp of the task without the need for fine-tuning. This would be an effective way to utilise these powerful services without sharing sensitive information. We will also explore the use of these synthetic reports for benchmarking our domain-specific models against general-purpose LLMs in zero-shot or few-shot settings. This will help us to assess their practical utility in real-world clinical information extraction.

Although the improvement was not statistically significant, incorporating additional contextual information into the model inputs yielded slightly better results. A more thorough analysis of prompt design will be conducted to determine whether further enhancing the model’s contextualisation can improve its performance; for example, using knowledge-driven prompts [27]. Finally, with a larger dataset, we will study the effectiveness of BioGPT in labelling multiple tumours within a single medical report.

## Data Availability

The datasets presented in this article are not readily available because the data was obtained under the research project PID2019-110686RB-I00, with the ethics approval from the HM Hospitals. To be used in the research project, but not to share, even if anonymised. Requests to access the datasets should be directed to mcarrilero@dia.uned.es.

## Footnotes

^1^https://www.cancer.org/cancer/breast-cancer/understanding-a-breast-cancer-diagnosis/breast-cancer-survival-rates.html, retrieved on 20/07/2025

## Appendix

### 1 Model parameters

The following parameters were selected during the cross-validation experiment. All the models have a batch size of 16 and a weight decay of 0.05.

The BoW-based neural network had an initial learning rate of 0.005 with an exponential decay rate of 0.98 and 60 decay steps (1 per 5 epochs) and was trained for 150 epochs.

The BERT-like models were fine-tuned first in the last classification layers for 5 epochs with a learning rate of 0.01. Table A1 show the parameters for the rest of the models and the second phase of the BERT-like models.

**Table A1:** Model parameters.

Model	Learning rate	Epochs
BlueBERT class.	$5\times 10^{-5}$	8
BlueBERT extr.	$1\times 10^{-4}$	7
BioBERT class.	$5\times 10^{-5}$	5
BioBERT extr.	$5\times 10^{-5}$	6
BioMedBERT class.	$5\times 10^{-5}$	8
BioMedBERT multi output	$5\times 10^{-5}$	7
BioMedBERT two-stage	$5\times 10^{-5}$, $3\times 10^{-5}$	3, 5
BioMedBERT extr.	$5\times 10^{-5}$	6
ClinicalT5	$7\times 10^{-5}$	7
BioGPT	$1\times 10^{-5}$	7
BioGPT two-stage	$1\times 10^{-5}$, $1\times 10^{-5}$	2, 7

Class. and extr. are abbreviations for classification and extractive.

Two-stage models have two learning rates and two epochs, one for each stage.
