# Supplementary material for: Extracting structured data from unstructured breast imaging reports with transformer-based models
Source: Front Digit Health. 2026 Jan 9;7:1718330. doi: 10.3389/fdgth.2025.1718330 (PMC12827707; doi:10.3389/fdgth.2025.1718330)
Supplement: Supplementary file 1 [file Datasheet1.pdf]

Supplementary Material

1 SUPPLEMENTARY DATA

Table S1. Confusion matrix for the BioGPT test experiment on the report technique. Rows correspond to ground truth classes and columns to predicted outcomes.

true/predicted	mammography and ultrasound	ultrasound	total
mammography and ultrasound	53		53
ultrasound		17	17

Table S2. Confusion matrix for the BioGPT test experiment on the report type. Rows correspond to ground truth classes and columns to predicted outcomes.

true / predicted	biopsy	nodal staging	normal	total
biopsy	5			5
nodal Staging		1		1
normal	1		63	64

Table S3. Confusion matrix for the BioGPT test experiment on the family history. Rows correspond to ground truth classes and columns to predicted outcomes.

true / predicted	no history	first	second	third	total
no history	49				49
first		6	1		7
second			7		7
third			1		1

Table S4. Confusion matrix for the BioGPT test experiment on the prosthesis. Rows correspond to ground truth classes and columns to predicted outcomes.

true / predicted	no	yes	total
no	62		62
yes		2	2

Table S5. Confusion matrix for the BioGPT test experiment on the BI-RADS. Rows correspond to ground truth classes and columns to predicted outcomes.

true / predicted	0	1	2	3	4A	4B	4C	total
0	1							1
1		9						9
2	1		33					34
3				7				7
4A					8			8
4B						3		3
4C							2	2

Table S6. Confusion matrix for the BioGPT test experiment on the mammography density. Rows correspond to ground truth classes and columns to predicted outcomes.

true / predicted	A	B	C	D	unknown	total
A	1					1
B		19				19
C			26			26
D				6		6
unknown					12	12

Table S7. Confusion matrix for the BioGPT test experiment on the calcifications in mammography. Rows correspond to ground truth classes and columns to predicted outcomes.

true / predicted	no	yes	total
no	34		34
yes	1	29	30

Table S8. Confusion matrix for the BioGPT test experiment on the lymph nodes in mammography. Rows correspond to ground truth classes and columns to predicted outcomes.

true / predicted	no	yes	total
no	61		61
yes	1	2	3

Table S9. Confusion matrix for the BioGPT test experiment on the ultrasound density. Rows correspond to ground truth classes and columns to predicted outcomes.

true / predicted	fib. and fat	het. fib.	homo. fatty	homo. fib.	unknown	total
fib. and fat.	5					5
het. fib.		24				24
homo. fatty			2			2
homo. fib.				18	1	19
unknown					14	14

fib., het. and homo. corresponds to fibroglandular, heterogeneous and homogeneous

Table S10. Confusion matrix for the BioGPT test experiment on the benign lymph nodes in ultrasound. Rows correspond to ground truth classes and columns to predicted outcomes.

true / predicted	no	yes	total
no	55		55
yes	4	5	9

Table S11. Confusion matrix for the BioGPT test experiment on the suspicious lymph nodes in ultrasound. Rows correspond to ground truth classes and columns to predicted outcomes.

true / predicted	no	yes	total
no	62		62
yes	1	1	2

Table S12. Confusion matrix for the BioGPT test experiment on the simple cysts in ultrasound. Rows correspond to ground truth classes and columns to predicted outcomes.

true / predicted	no	yes	total
no	33		33
yes		31	31

Table S13. Confusion matrix for the BioGPT test experiment on the ductal ectasia in ultrasound. Rows correspond to ground truth classes and columns to predicted outcomes.

true / predicted	no	yes	total
**no**	60	1	61
**yes**		3	3

Table S14. Confusion matrix for the BioGPT test experiment on the nodules in ultrasound. Rows correspond to ground truth classes and columns to predicted outcomes.

true / predicted	no	yes	total
**no**	37	2	39
**yes**	2	23	25

Table S15. Confusion matrix for the BioGPT test experiment on the shape descriptor in ultrasound. Rows correspond to ground truth classes and columns to predicted outcomes.

true / predicted	irregular	lobulated	oval	unknown	total
**irregular**	1				1
**lobulated**		7			7
**oval**		1	3		4
**unknown**		2		11	13

Table S16. Confusion matrix for the BioGPT test experiment on the margin descriptor in ultrasound. Rows correspond to ground truth classes and columns to predicted outcomes.

true / predicted	circumscribed	indistinct	not circumscribed	unknown	total
**circumscribed**	11				11
**indistinct**		1		1	2
**not circumscribed**			1		1
**unknown**				11	11

Table S17. Confusion matrix for the BioGPT test experiment on the echogenicity descriptor in ultrasound. Rows correspond to ground truth classes and columns to predicted outcomes.

true / predicted	complex cystic	hypoechoic	isoechoic	unknown	total
**complex cystic**				1	1
**hypoechoic**		12			12
**isoechoic**			2		2
**unknown**				10	10

Table S18. Confusion matrix for the BioGPT test experiment on the previously known nodule or not. Rows correspond to ground truth classes and columns to predicted outcomes.

true / predicted	no	yes	total
**no**	10	2	12
**yes**	3	10	13

Table S19. Confusion matrix for the BioGPT test experiment on the stability of the known module. Rows correspond to ground truth classes and columns to predicted outcomes.

true / predicted	grown	stable	total
**grown**	1	1	2
**stable**		11	11
